# 3D Textiles Based on Warp Knitted Fabrics: A Review

**DOI:** 10.3390/ma16103680

**Published:** 2023-05-11

**Authors:** Lars Hahn, Konrad Zierold, Anke Golla, Danny Friese, Steffen Rittner

**Affiliations:** 1 Institute of Textile Machinery and High Performance Material Technology (ITM), Technische Universität Dresden (TU Dresden), 01069 Dresden, Germany; 2 Structural Concrete Institute, Leipzig University of Applied Sciences (HTWK Leipzig), 04277 Leipzig, Germany

**Keywords:** warp knitting, 3D textiles, spacer fabrics, preforming, non-crimp fabrics

## Abstract

Fibre-reinforced composites (FRCs) are already well established in several industrial sectors such as aerospace, automotive, plant engineering, shipbuilding and construction. The technical advantages of FRCs over metallic materials are well researched and proven. The key factors for an even wider industrial application of FRCs are the maximisation of resource and cost efficiency in the production and processing of the textile reinforcement materials. Due to its technology, warp knitting is the most productive and therefore cost-effective textile manufacturing process. In order to produce resource-efficient textile structures with these technologies, a high degree of prefabrication is required. This reduces costs by reducing the number of ply stacks, and by reducing the number of extra operations through final path and geometric yarn orientation of the preforms. It also reduces waste in post-processing. Furthermore, a high degree of prefabrication through functionalisation offers the potential to extend the application range of textile structures as purely mechanical reinforcements by integrating additional functions. So far, there is a gap in terms of an overview of the current state-of-the-art of relevant textile processes and products, which this work aims to fill. The focus of this work is therefore to provide an overview of warp knitted 3D structures.

## 1. Introduction

At the United Nations Climate Change Conference COP27 in 2022, participating countries reaffirmed their commitment to limit the global temperature increase to 1.5 degrees Celsius above pre-industrial levels [1]. This means implementing solutions to reduce emissions of greenhouse gases such as CO_2_.

At the same time, the world’s population is expected to grow to more than 10 billion people by 2100 [2]. Accordingly, greenhouse gas emissions will increase if energy and resource consumption per person remains unchanged. Therefore, a drastic action is needed to reduce the global demand for energy and resources in order to meet the 1.5 degree Celsius target.

Achieving environmental sustainability through the use of innovative materials is a major technology driver and an effective response to resource depletion and the need to drastically reduce CO_2_ emissions [3,4,5]. The cross-industry use of fibre-reinforced composites (FRCs) can be a decisive key [6]. For example, in the automotive and aerospace sectors, the main advantage of using FRC compared to metallic materials is that moving masses are reduced. This reduces the energy requirement and thus CO_2_ emissions. FRCs consist on the one hand of a fibre reinforcement and on the other hand of a polymer or mineral matrix surrounding the fibre reinforcement [7]. Their anisotropic structural-mechanical properties, which can be tailored compared to metallic materials and their low specific weight create the best conditions for the resource-efficient design and implementation of lightweight construction solutions [7,8].

FRCs are key innovation drivers in the fast-growing and future-oriented market segments of renewable energy (e.g., for wind turbines and high-pressure vessels), electromobility, and aerospace. Lightweight construction is also of particular importance in mechanical and plant engineering [9,10,11,12,13,14,15,16].

Textile semifinished products usually need to be draped to form a preform suitable for production of FRC components. In particular, the preforming results in terms of wrinkle and overlap free forming of the NCF or buckling of spacer fabrics have a major impact on the cost of the FRC part, the utilisation of the performance of the textile reinforcement structure and the saving of resources by avoiding oversizing [17,18,19]. Even simple geometries suffer from fibre displacement during draping, resulting in gaps, overlaps and wrinkles. Due to the anisotropic properties of high-performance fibres (very high tensile stiffness with low compressive stiffness/strength), even small deviations in the fibre orientation from the nominal orientation cause a significant stiffness reduction in the FRC component (10° deviation, approx. 30% less stiffness). The result is a component oversizing [20,21,22].

It is concluded that a high degree of prefabrication, in the form of 3D textile structures, further enhances the economic and environmental benefits of using FRCs. Preform costs can be reduced, fibre cuttings can be saved and the utilisation of material performance can be increased [9,14,23,24,25,26,27].

Warp knitting is one of the most productive and cost-effective textile manufacturing processes due to its technology of simultaneously forming stitches across the entire working width of the machine, making it particularly interesting for industrial applications. Basically, the warp knitting process either creates a textile surface with the warp threads or the warp threads are used to fix additional reinforcement yarns [7,28,29].

The industrial implementation of 3D warp knitted structures is an important basis for increasing the economic efficiency and further reducing the environmental footprint of FRC. Reducing the cost of preforming is therefore the key parameter for increasing the industrial attractiveness of composites [30,31,32,33].

In this context, the use of warp-knitted FRCs with a high degree of prefabrication plays a key role in the economical and material-saving use of the limited resources available. Particularly important are multiaxial non-crimp fabrics (NCFs) produced on highly productive multiaxial warp knitting machines and warp knitted spacer fabrics produced on double bar warp knitting machines. Non-crimp fabrics are characterised by the fact that the reinforcing yarns are stretched (no crimp), allowing forces to be absorbed directly by this type of fabric in the FRC without structural stretching.

Significant quantities of multiaxial fabrics are already in use today, particularly for large-scale applications (e.g., automotive), large area components (e.g., wind rotor blades) and highly stressed aerospace FRC components (e.g., fuselage segments) [7,34]. Warp knitted spacer fabrics are mainly used for pressure relief, heat regulation and fluid transport applications [35,36,37,38].

The review provides an overview of the manufacturing requirements, textile production methods and process limitations for the implementation of 3D warp knitted fabrics. Possibilities for varying the 3D appearance through material, structure and process changes are discussed in detail.

The paper is a guide for a wide range of readers, from fibre and textile scientists to those involved in the research and development of new generation 3D textile products, especially warp knitted structures.

## 2. Method and Systems

### 2.1. Method

In order to assess the state of scientific and technical knowledge in the field of 3D textiles, with a focus on warp-knitted three-dimensional fabrics, an investigation was carried out. The state-of-the-art and research was investigated, based on the literature and patent search as well as interviews with experienced textile technologists. Accordingly, an overview of warp knitted three-dimensional fabrics, in particular 3D non-crimp fabrics and warp knitted spacer fabrics is given. The focus of the presentations is on the documentation of existing machine technologies and their current technological limitations, products already implemented and their applications.

The research results provide a basis for the derivation of suitable basic technologies for future developments. The knowledge gained also enables the derivation of future research fields.

### 2.2. 3D Warp Knitted Fabric Classification

There are two different approaches to the realisation of 3D warp knitted fabrics, these include non-crimp fabrics (NCFs) and spacer fabrics. Focusing on the draping process of these warp knitted fabrics, the subdivision is as follows:

A: subsequent draped. Wrapping a component contour with warp knitted fabrics without wrinkles or distortion is made possible by the sliding integration of the reinforcing yarns, which can slide past each other during the draping process until the required yarn length distribution is achieved, and also in combination with the high elasticity of the warp knitting yarn.

B: direct draped. This approach provides for the targeted predefinition of reinforcing yarn lengths in warp and weft directions for wrinkle- and distortion-free wrapping of or over a component contour with warp knitted fabrics in the production process, in order to save the subsequent forming process described in A.

**Figure 1:**
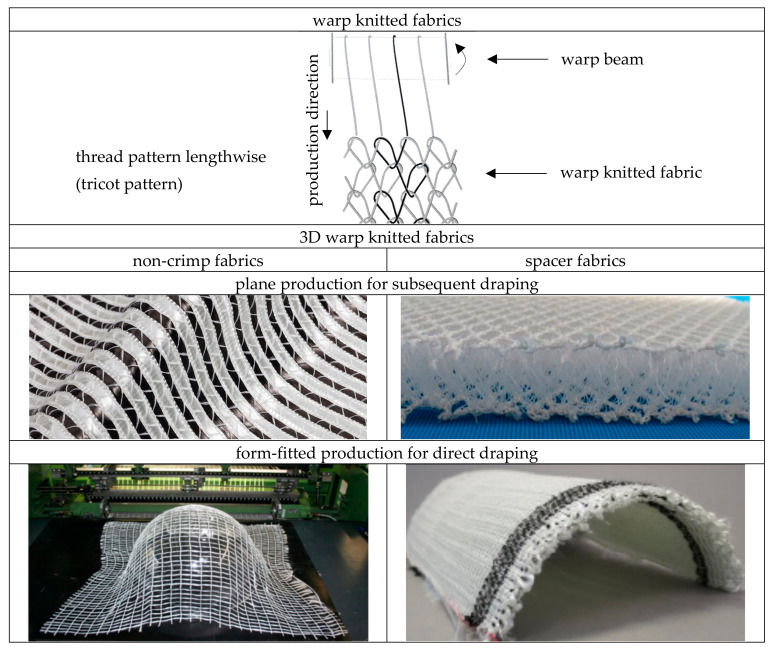
Classification of warp knitted fabrics for 3D reinforcements [7,18,31,31,38,39,40,41].

An overview about the classification of warp knitted fabrics for 3D reinforcements is given in Figure 1. When comparing NCF and spacer fabrics, spacer fabrics already have a 3D structure after machine production due to the additional pile yarns formed in the spatial or Z-direction (see also Figure 5). Details of pile yarns are given in Section 2.3.2.

Due to the special spacer structure of the warp knitted spacer fabric, which creates hollow chambers, there are other applications than NCF, such as medical filters for dialysis applications.

**Figure 2:**
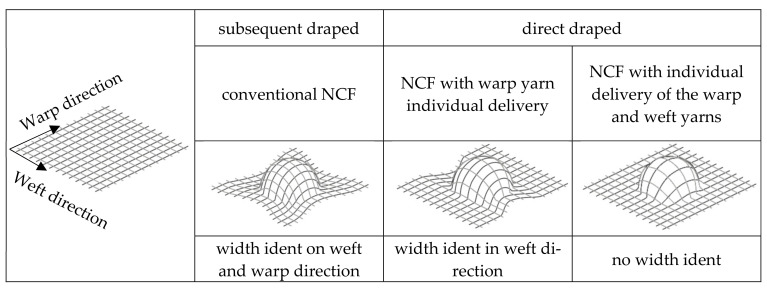
Principle—subsequent and direct draping of preforms from non-crimp fabrics according to [31,33,42,43,44].

Figure 2 illustrates the different processes in the draping of preforms especially made of NCF produced by the direct preforming process, with focus on the different lengths of the warp and weft yarns.

### 2.3. Technological Approaches to the Production of 3D Warp Knitted Fabrics

#### 2.3.1. Warp Knitting for 3D Non-Crimp Fabrics

**Figure 3:**
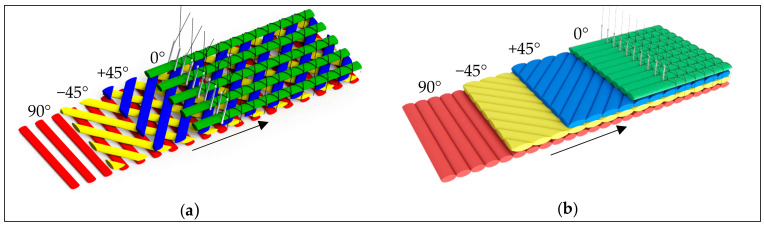
Exemplary production principle of 2D multiaxial warp knitted structures (**a**) lattice-like non-crimp fabric, (**b**) (closed non-crimp fabric) according to [7,48,49].

The established basic process for the production of 3D NCF is the multiaxial warp knitting technique [7,45,46,47]. Compared to other processes such as weaving, multiaxial warp knitting is characterised by the particularly high productivity of the laying and knitting technology. Depending on the requirements, the weft yarns are hooked into the transport chain, typically in a 90°, +45° or −45° direction, in a stretched orientation, see Figure 3.

The weft yarns move in a continuous, translatory motion to the knitting point. At the knitting point the 0° yarn layer is inserted. The layers of yarn are then fixed with a knitting or binding yarn by the stitch process (warp knitting process). In principle, it is possible to produce lattice-like and closed non-crimp fabrics (NCF) [7].

**Figure 4:**
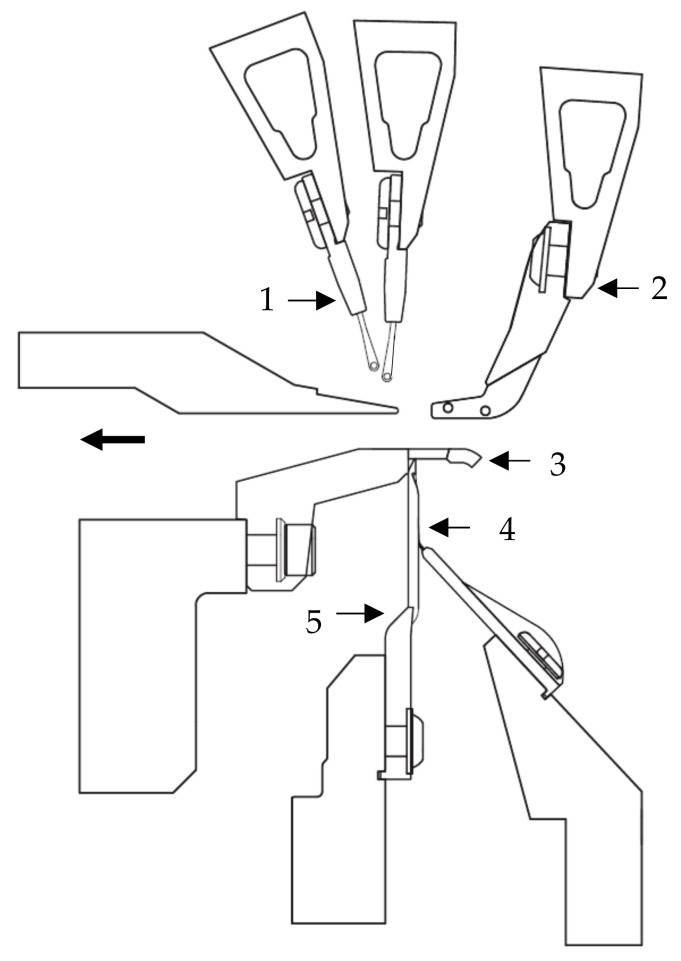
Knitting elements of an exemplary warp knitting machine with multiaxial weft insertion (1: Guide bar with guide needles, 2: Pillar thread sinker, 3: Trick sinker, 4: Closure, 5: Compound needle, production direction right to left) according to [7,52,53].

The looping of the yarn plies with the warp knitting thread in the stitch formation process provides a basic structural stability to the textile structure. After the knitting process, the weft yarns of the textile surface are cut or needled out from the transporting warps and wound onto a fabric beam. Figure 4 schematically shows the knitting point of a multiaxial warp knitting machine with the corresponding working elements.

Due to the straight and ondulation-free orientation of the yarn layers, structural elongation is reduced to a minimum. Therefore, in addition to the numerous applications in the automotive, aerospace and sporting equipment industries, scrim structures are particularly suitable for use in the construction industry, for reinforcing the brittle material concrete [7,50]. The critical concrete strain for normal strength concrete is εcu1 = 3.5% [51]. If this value is exceeded in structures, the consequence is that the serviceability limit state is exceeded. Therefore, a textile structure is required that has the lowest possible structural strain when loaded. This will prevent the development of large cracks when the textile concrete is loaded, thus ensuring serviceability.

#### 2.3.2. Warp Knitting for Spacer Fabrics

**Figure 5:**
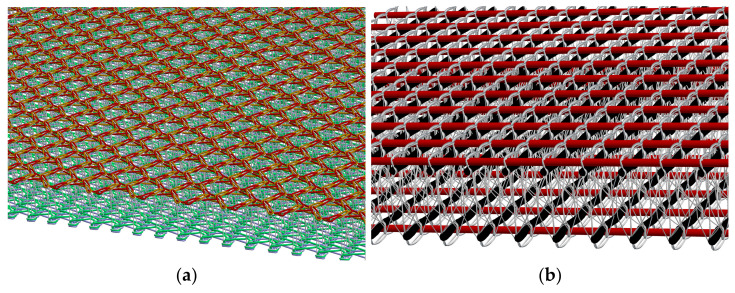
Planar warp knitted spacer fabrics without (**a**) and with (**b**) 0°—(black dyed yarns) and 90°—(red dyed yarns) reinforcing yarns (pile yarns connected both sides of the fabric).

The machine technology enables the production of two opposite arranged RL warp knitted fabrics, which form the surface layers of a warp knitted spacer fabric and that can be joined in z-direction by a third thread system, see Figure 5.

The so-called pile yarns be inserted into both sides of the fabric sides to connect them in defined densities. The maximum spacing of warp knitted spacer fabrics depends on the maximum distance between the trick plates. Currently, machine spacing settings for the production of spacer fabrics are realised to up to 65 mm [38,54].

**Figure 6:**
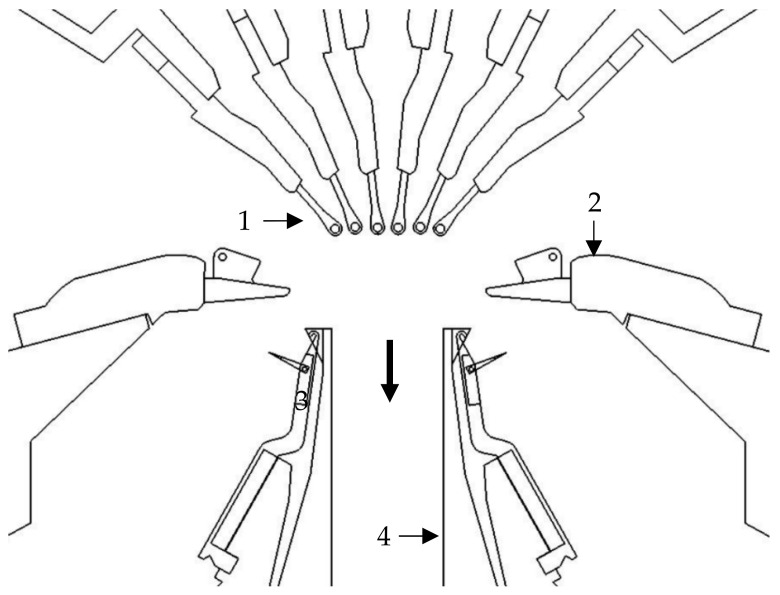
Knitting elements of the double needle bar raschel machine (1: Guide bar with guide needles, 2: Stitch-comb bar, 3: Needle bar with latch needles, 4: Trick plate, production direction from top to bottom) according to [38,55].

Warp knitted spacer fabrics are generally produced on double needle bar raschel machines also known as double needle bed raschel machines, as shown in Figure 6.

The high pattern variation range of warp knitting technology allows the pressure stability and air permeability of spacer fabrics to be specifically adjusted. As a result, warp-knitted spacer fabrics are commonly used in seating, automotive, functional clothing, mattresses, medical support textiles such as bandages and orthopaedics. Their sideways open structure provides elasticity, insulation and acoustic damping properties as well as being used for filtration. Other technical applications are therefore currently in the field of filtration systems and water harvesting methods.

There are also jacquard double bar raschel machines capable of producing (dendritic) tubular structures and spacer fabrics [56]. The production of 3D NCF based on warp knitted spacer fabrics requires the integration of warp yarns inserted as 0° warp yarns without stitches and weft yarns. This requires a double needle bar raschel machine with a double faced weft insertion system. The integration of high-performance yarns, such as glass yarns, on both sides in 0° and 90° directions currently allows the production of open textile-reinforced, spacer warp knit mesh structures for concrete applications (Figure 14), such as façade elements (Figure 17) and bridge elements [57].

## 3. Textile Technologies for Production of 3D Warp Knitted Fabrics

### 3.1. Warp Knitted 3D Non-Crimp Fabrics

Non-crimp fabrics (NCFs) are mainly made from homogeneous 2D textile structures in the form of rolls with constant yarn spacing and widths [17].

The manipulation of a 2D NCF to create the desired shape or form is called draping for the processing of 3D fabrics. This is typically done by laying out the 2D NCF and then folding, pleating, or otherwise manipulating it to achieve the desired shape. As a basis for composites fabrication, the resulting shape is then pinned, sewn or impregnated to hold it in place [7].

The underlying mechanism of draping is the stretching and compression of the warp knitted fabric. When the fabric is manipulated, by applying forces to the outer edges, it is stretched in some areas and compressed in others, creating a pattern of tension and compression across the material (see Figure 2). The effects of draping on textiles have important implications for the manufacture of fibre reinforced composites (FRCs).

NCF can be divided into three categories according to their three-dimensional configuration and shaping. Material (a): Binding and reinforcing yarn densities are constant, draping due to lack of structural stretch in the warp knitting thread [58]. Structure (b): Constant binding and variable reinforcing yarn density and orientation [31,33]. Process (c): Variable binding (by adhesive or warp knitting thread) and constant reinforcing yarn density and orientation [34,56].

The advantages and disadvantages of each class in terms of their applicability to complex three-dimensional composite components are discussed below. There is no known research into the use of a combination of variable binding, variable reinforcement yarn density and orientation.

#### 3.1.1. Variation through Material and Structure Modification

##### Material

In this category, there are two options for the subsequent drape of the NCF. Option 1: The differences in warp knitting thread lengths required for a draping process are achieved through thread shifting during the draping process. Option 2: The differences in thread orientations required for a draping process are achieved through stretching and twisting of the warp knitting thread during the draping process from 2D to 3D.

**Figure 7:**
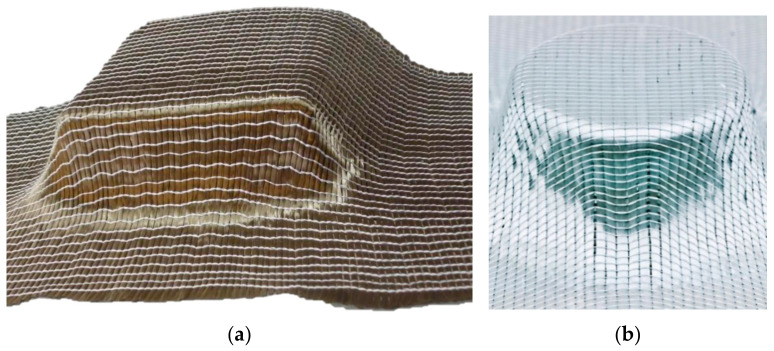
Representation of the DRAPETEX/DRAPEFIX, subsequent-drapable non-crimp made of basalt (**a**) and glass and carbon fibres (**b**) from the company Gerster TechTex (Germany) [58,60].

An example for draping due to lack of structural stretch of the warp knitting threads is the NCF with partial weft fringe binding “Drapetex” from the company Gerster TechTex (Biberach an der Riß, Germany), see Figure 7. NCF with material variability are neither close to contour nor a preform, and orientation of the reinforcing yarns according to the load path is not possible. Such NCF can be produced on conventional multiaxial warp knitting machines without further development and adaptation of the machine technology. Depending on the complexity of the part, subsequent draping of such an NCF can produce a wrinkle-free and gap-free fabric surface over the preform surface. However, this results in unavoidable wrinkles and gaps outside the preform surface and therefore unavoidable waste. In addition, it is not possible to orient the reinforcement yarns (weft and warp) during the draping process in accordance with the load [58,59].

##### Structure

**Figure 8:**
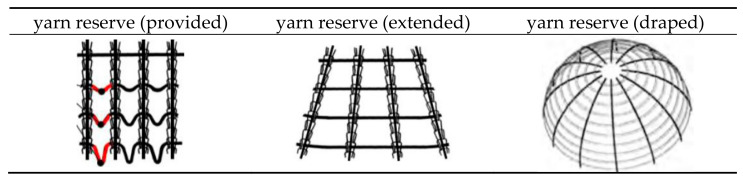
Illustration of the principle of formation of additional yarn length sections in near net shape non-crimp fabric for better draping behaviour using the example of weft yarn length sections between warp yarns to achieve of wrinkle free draped non-crimp fabric according to [31,42,44,61,62].

Multiaxial warp knitted fabrics are basically composed of three yarn systems. 1. The warp knitting yarns (threads), 2. The warp yarns and 3. The weft yarns. For all and within all three yarn systems, forces act on the yarns to induce stretching and wrinkling when the NCF is draped. Therefore, one starting point for producing NCF with a high degree of prefabrication is to determine the forces/stresses that occur prior to NCF production and to design the yarn systems accordingly so that the forces/stresses that occur during draping are minimal. In theory, this means that wrinkles, overlaps and gaps in the preform can be avoided without the need for structural over-dimensioning, e.g., through additional yarn layers, in order to compensate for these defects. Since NCF made from high-performance fibres, such as carbon or glass, have only a low material ductility, even relatively low draping forces lead to large displacements of the yarn layers. A promising approach to compensate for the different draping forces in NCF is to give each yarn a pre-defined individual length based on the final three-dimensional shape in the draped state, hereafter referred to as the yarn reserve [31,32,33,42,43,61]. These yarn reserves may be unevenly distributed over the yarn laid down (inhomogeneous yarn reserve) or evenly distributed over the length of yarn laid down (homogeneous yarn reserve). A schematic representation of the behaviour of yarn reserves in the draping process of near net shape NCF is given in Figure 8.

A homogeneous yarn reserve is based on the compensating for the different forces during draping by feeding yarn material from the components that were not originally in the preform area. This overlaps with the material approach.

The principle of inhomogeneous yarn reserve is based on the work of Sankaran et al. [31], where yarn reserves are created in defined subsections of the warp reinforcement yarns. These yarn reserves correspond to the yarn section lengths required for subsequent draping. Based on this, a technological approach for the additional creation of a weft reserve was developed in further research at the TU Dresden [33,42]. The two developments approaches for creating an inhomogeneous warp and weft reserve are described below. Figure 2 gives an overview of the basic geometric of planar NCF to 3D NCF preforms with varying warp and weft yarn length.

##### Structure—Warp Yarn Reserve (0°-Direction):

**Figure 9:**
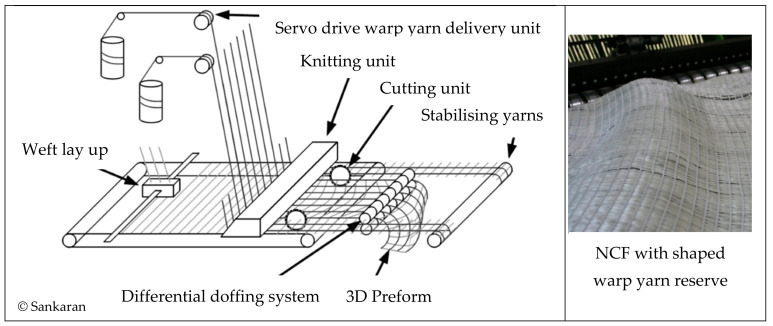
Schematic illustration of how the warp reserve works on a multiaxial warp knitting machine (**left**) and pattern structure (**right**) according to [31,32,43].

In the field of technical textile production, warp reinforcement yarns are provided on bobbins. Warp beams for reinforcement yarns are not known. Since each yarn comes from its own pay-off point, there is basically the possibility of warp yarn reserve. The stitch formation process is discrete in time. The warp thread moves at a constant speed, independent of the stitch formation process, if it is wound up with a constant tension force. If the take-off speed/take-off force is changed in a warp thread-specific manner, the distances between the stitches in the formed fabric are changed in the warp direction (directly proportional). In this way, warp yarn reserves can be generated. The change in take-off force can be achieved, for example, by means of a single-yarn take-off or a mechanical shaping located between the take-off and the knitting point. The single yarn take-off (in combination with a single yarn delivery to reduce the required take-off forces at the differential doffing system) was developed technologically and structurally by Sankaran et al., and a schematic representation of the functioning of this principle is shown in Figure 9. It was implemented on a KARL MAYER Malimo 14024 multiaxial warp knitting machine manufactured by KARL MAYER Technische Textilien GmbH (Chemnitz, Germany).

Technical limitations are the length of the warp reserve (in Sankaran’s implementation, the excess length factor was 1.5) [31]. However, without a change in weft spacing and weft lengths, crease-free draping is not possible with warp reserve alone [42]. Width incursion transverse to the warp direction, caused by the weft yarns of different lengths, remains. Since the number of yarns fed per unit of width (warp yarns) and per unit of length (weft yarns) remains constant over the working width or in the weft lay-up, different material requirements (in this case number of yarns/density) caused by 3D shaping/contouring cannot be compensated, so that local deviations in the basis weight within the NCF inevitably occur. The ITM of TU Dresden is currently developing solutions for inline manipulation of warp density (final contour-optimised NCF). A research project on the manipulation of weft density is planned for the end of 2023.

Fundamental research activities by Sankaran et al. led to the development of curved reinforcing grids that can be specifically adapted to geometric shapes through variable single warp yarn delivery [63]. The application of this technological approach is limited to simple 3D contours. In the case of complex component shapes, significant, undesirable deflections of the reinforcing filament course (structural distortions) occur, leading to a reduction in the load-bearing capacity in the later component [31].

In the case of complex component shapes, significant, undesirable deflections of the reinforcing yarn course (structural distortions) occur, which lead to a reduction in the load-bearing capacity in the later component. Semi-finished textile products that meet the requirements and can be precisely adapted to a given component geometry can only be produced if the lengths of both the warp and weft sections can be flexibly varied locally between the bonding points [33,42].

##### Structure—Weft Reserve (90°-Direction):

**Figure 10:**
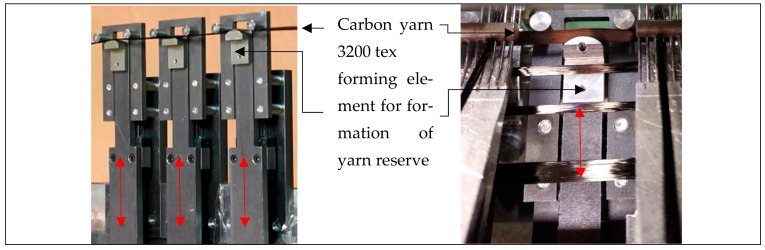
Technological approach to forming a weft yarn reserve on a test rig (**left**) and built into the multiaxial warp knitting machine of the type KARL MAYER Malimo 14024 (**right**), of the KARL MAYER Technische Textilien GmbH (Germany) according to [33,42,61].

In all known methods for forming the inhomogeneous weft reserve, the yarn reserve is formed mechanically, i.e., by means of mechanical forming tools. A technological approach to forming a weft yarn reserve is shown in Figure 10.

The limitations of mechanical forming of the yarn reserve lie in the space required for the forming elements and the forming movement. As a result, only lattice structures can effectively be produced if the high productivity of multiaxial warp knitting technology is to be maintained. Geometric design is therefore limited in this respect (for a hemispherical preform, for example, the maximum radius that can be converted is 0.64 × working width) [33,42]. The time of the width indent is therefore shifted directly into the manufacturing process. As a result, the NCF produced already has a variable outer contour after production, even before draping. Furthermore, gaps are built into the fabric during weft yarn formation. Rather than increasing the material requirement of the fabric “from the outside”, the spacing between the yarns is adjusted. This reduces the yarn density/area mass in the drape area. Therefore, inhomogeneous yarn reserves can only be used sensibly if they are combined with measures for force-flow conformity (fibre follows force).

The advantage of the yarn reserve principles is that it is easier to automate than draping, e.g., Drapetex. The semi-finished textile product must be precisely positioned in the mould in two planar coordinates and one angular coordinate. There is no need to apply additional forces to move and stretch the threads. The automation technologies therefore need to be less complex than for conventional 2D textiles or material-based drapability optimisation.

#### 3.1.2. Variation through Process Modification

**Figure 11:**
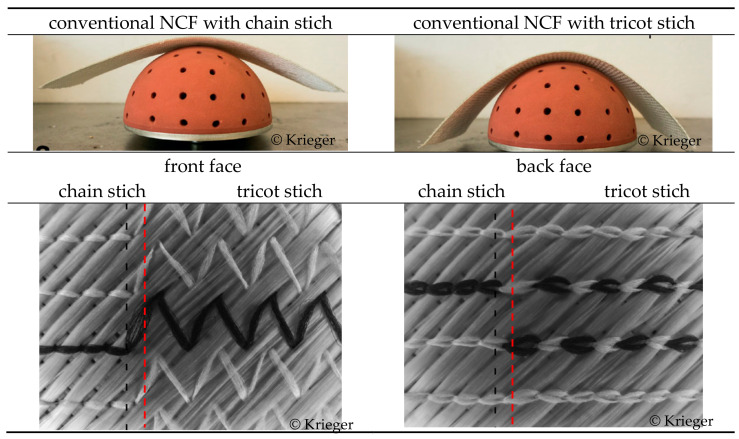
Technological principle for tailored non-crimp fabrics according to [18].

Investigations by Krieger et al. show approaches to influence the draping behaviour of NCF by changing the binding type of the warp knitting thread in the NCF during the production process [18]. The so-called tailored non-crimp fabrics (TNCFs) have been developed to achieve sufficient drapability or low shear stiffness for forming into double-curved geometries on the one hand and high dimensional stability or high shear stiffness for automated handling on the other hand. As a result, they developed a TNCF with a stitch length of 2 mm, a stitch gauge of E6, and a change in stitch type from chain to tricot, see Figure 11.

An alternative stitch-free approach was taken by Rittner et al. by replacing the knitting yarn with a chemical binder, solidified by heat and pressure, in combination with a CAE-simulation to investigate the optimal binding in a two- or four-layer NCF, depending on the drape geometry [34,42]. The freedom in shaping is not yet as great as with the aforementioned approaches (compare Figure 9), but a combination of this process with the structure approach potentially offers possibilities for generating force-flow-oriented textiles with a high degree of prefabrication. However, accurate positioning on the mould is a prerequisite for obtaining a draped fabric free of wrinkles and warping. This requires a clear positioning rule, e.g., by marking points on the NCF, which results in increased demands on the entire production process chain.

### 3.2. Warp Knitted Spacer Fabrics

According to Hu et al., the advantages of three-dimensional warp knitted spacer fabrics are as follows [30]:

- Inexpensive and easy to manufacture;
- Handling of preforms layers prevented from moving through stitching;
- Better impact damage tolerance;
- Improved delamination resistance to ballistic and blast loads;
- Improved interlaminar fatigue resistance;
- Improved joint strength under monotonic and cyclic loading.

#### 3.2.1. Variation through Material and Structure Modification

**Figure 12:**
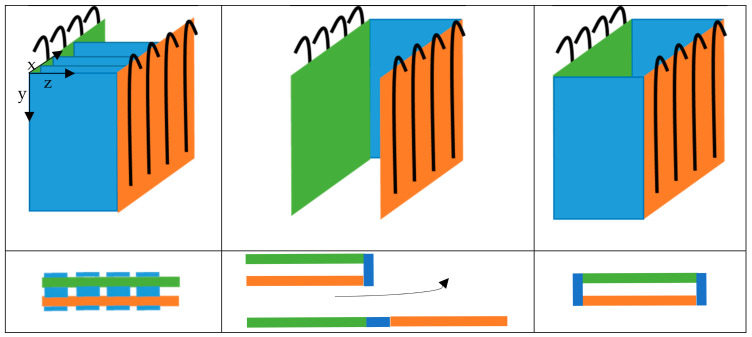
Schematic representation of the basic structures of warp knitted spacer fabrics, according to [64].

There are several textile technological options for the production of warp knitted spacer fabrics, an overview of the basic structures is given in Figure 12.

In order to achieve a straight yarn orientation of reinforcing yarns, they must not form a mesh by themselves. Therefore, each reinforcement yarn system must be held in place by a warp knitting thread. The following explains how the reinforcement yarn layers can be integrated into the spacer fabric in the x, y and z directions based on the double needle bar warp knitting technology.

In order to achieve a straight reinforcement yarn position across the width in the X-direction, defined as the working width direction of the warp knitting machine (or 90°-direction), it is necessary to integrate weft yarns, e.g., in the form of a long weft or magazine weft. A warp knitting thread system with a pillar stitch is a suitable structure for binding these weft yarns in the spacer warp knit. It is also possible to integrate segmented straight yarn sections across the working width by means of a partial weft structure known as inlay warp knitting. The pillar stitch binding is also suitable for holding the inlay yarns in place [64,65].

The integration of a reinforcing layer in the Y-direction can be achieved either by a partial weft lay staggered over a needle lane and combination of elastic and inelastic yarn material. The Y-direction, or 0°-direction, is defined as the production direction. The Y-thread has to consist of low elasticity material that has to be drawn to a straight thread. The yarn supply is therefore low. The low-elasticity material yarn needs to be bound with an additional loop yarn system (e.g., tricot or atlas binding) consisting of an elastic yarn. Another way of integrating straight yarns in the y-direction is to use filler yarns, which are located and remain in a needle lane. They can be bound either by a weft yarn system in front of them or in combination with a loop thread system in front of them, e.g., by a pillar stitch, or the filler yarn can be integrated between two yarn systems of the outer surfaces, forming crossed wales (e.g., tricot binding) [64,65].

Pile yarns present in warp knitted spacer fabrics can also have a reinforcing function in the Z-direction (thickness direction). Basically, the shear strength, compressive strength and thickness of the spacer fabric are determined by the material used and the warp knitting integration of the pile yarn. The pile yarns can be placed in between the textile cover surfaces in a straight form. It is essential that the pile yarns are crimped in the area of the surface layers to enable fixation there. In general, the pile yarns can be fixed within the textile face layers by open or closed loops [64,65].

**Figure 13:**
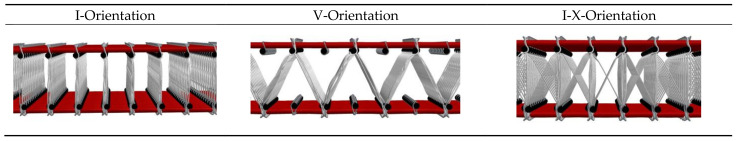
Pole thread orientation options in warp knitted spacer fabrics.

The binding of the pile yarn system defines the orientation of pile yarns in the cross section of the spacer warp knit. A possible structure for the pile yarns with a straight orientation is in a 90° I-orientation, realised with a pillar stitch lapping in pile yarn system and at least one thread system. Another option is to incorporate the pile yarns at a defined angle (V-orientation), depending on the yarn threading and machine gauge, as well as the lapping as tricot/cord/atlas. This can be implemented with one or preferably with two thread systems. Another option is the X-shaped binding of the pile yarns in the spacer fabric cross-section. This is realised by 2 pile yarn systems and a counter lapping of a tricot, cord or atlas lapping [64,65]. The highest shear strength is achieved with the combination of I and X-shaped binding of the pile yarns in the spacer fabric cross-section. Figure 13 presents an overview of existing orientation possibilities for the integration of pole yarns in spacer fabrics.

3D reinforcement structures made of warp-knitted spacer fabrics have been used for concrete reinforcement in recent years [66,67]. 3D warp-knitted spacer fabrics offer the advantage that two layers of reinforcement can be integrated into one textile and can be designed variably. This allows the degree of reinforcement to be freely adjusted via the mesh size. The high stiffness of the spacer yarn enables the reinforcement layers to be fixed to each other in a precise position, which is essential for reinforcing concrete.

**Figure 14:**
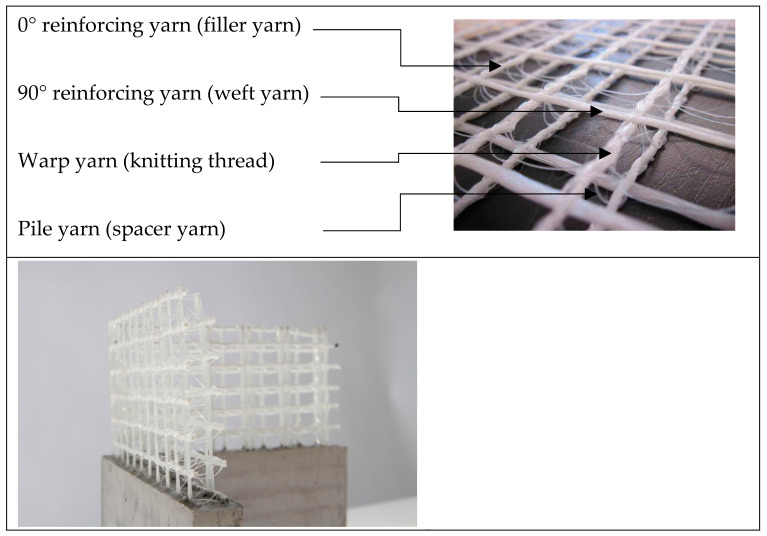
Example for a warp-knitted spacer fabric used for constructions applications [67,70,71].

The individual layers are similar to the two dimensional, lattice-like NCF described in Figure 14 and consist of reinforcing yarns in 0° and 90° directions and a warp yarn connecting them. The spacing between the layers can be adjusted during production [68,69].

Another application involving integration of functional materials in the z-direction is spacer-knitted thermoelectric generators (TEGs). Metal wires have been integrated as functional and reinforcing materials in different structures in z-direction, which can generate electric current from waste heat. The TEGs achieve an electrical power of 1.78 μW [72].

#### 3.2.2. Variation by Process Modification

In order to produce more complex spacer fabrics with additional spatial curvatures, machine modifications of the double needle bar warp knitting technology are necessary. In principle process modifications in the double needle bar warp knitting process and thus product changes can be achieved by (1) varying the trick plate spacing and position, (2) by varying the fabric take-off and yarn tension and (3) by integrating additional technologies.

An overview of the known technologies for the production of modified warp knitted spacer fabrics is given below.

**Figure 15:**
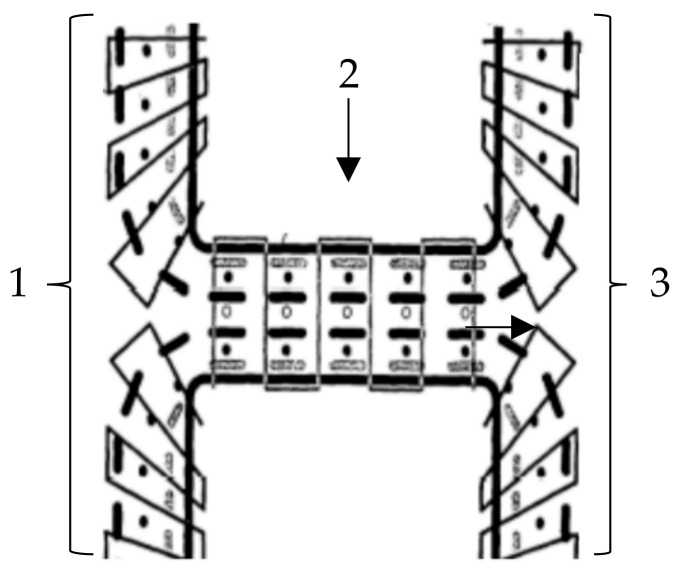
Textile semi-finished product for fibre-reinforced plastic profiles (1, 3: legs made of right-left multiaxial warp knits, 2: bar made of a right-right multiaxial warp knitted structure [73].

Figure 15 shows a textile semi-finished product for fibre-reinforced plastic profiles, based on the patent of Arnold and Hufnagl [73,74]. Such structures are preferably used for the production of T or double-T profiles. The bar of the textile profile is made from a right-right multiaxial warp knitted structure. The legs are made up of right-left multiaxial warp knits. Such warp knitted spacer fabrics are made possible by varying the spacing and position of the trick plates.

A further embodiment of the double needle bar warp knitting process is the use of a knitted fabric take-down with frictionally engaged conical take-down rollers [75]. This makes it possible to produce arc-shaped closed reinforcing knitted fabrics with in-plane curvature.

Further developed double bar warp knitting techniques also make it possible to produce spacer warp knits with alternating distances between the partial areas within the working width and thus contoured closed and/or lattice-like surfaces [76].

In parallel, research by Gries et al. looked at local reinforcement using inserts, for example for shell-stringer structures. For this purpose, a multiaxial warp knitting machine was modified, with which the production of the NCF and the connection with an already prefabricated textile stringer structure are carried out in a single operation [77]. However, these spacer warp knits do not allow for a curved reinforcing yarn path, requiring near net structures.

**Figure 16:**
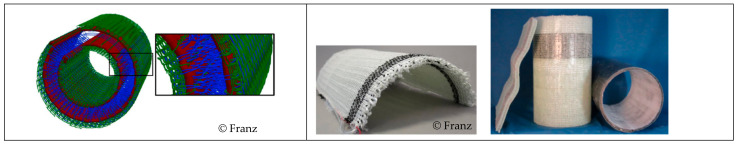
Tubular warp knitted spacer fabrics (left: simulation model, right: textile and consolidated demonstrator [38]).

Franz developed warp knitted spacer panels [78] and tubular warp knitted spacer fabrics [38], see Figure 16. Tubular warp knitted spacer fabrics are producible by varying the fabric take-off and yarn tension. Based on this technology, fibre-reinforced lightweight components can be developed in a highly productive manner for large-area components such as ceiling, wall or floor elements, as well as for double-walled pipes. Compared to conventional structures available on the market, such as honeycombs and glass spacer fabrics, the spacer structures produced in integral design have a six-fold higher compressive strength.

**Figure 17:**
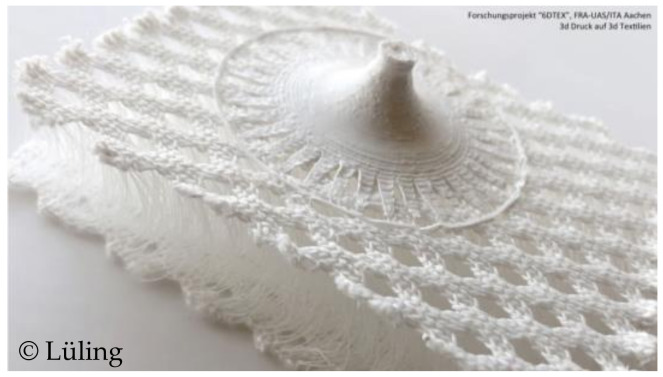
Warp knitted spacer fabric combined with a 3D-printed object for lightweight building components [81].

Lüling has developed a technology for lightweight building components by combining 3D spacer fabrics with 3D printed objects. Such products can be used, for example, as textile façade shells for targeted shading and insulation. A key focus in the development of such structures has been to use the same groups of materials for both the print and the textile material, making the recycling process easier [79,80]. An example of such a structure is given in Figure 17.

## 4. Conclusions and Outlook

The use of textiles with a high degree of prefabrication, such as 3D warp knitted fabrics, reduces the effort required in the post-textile production processes for the realisation of fibre-reinforced composite structures. It also enables innovative applications for textile structures. In particular, warp knitting technology is of considerable economic importance due to its principle-based high productivity.

The review shows that there are a variety of textile engineering approaches for the production of 3D warp knitted fabrics. The classification in this paper is made in terms of machine technology into multiaxial knitting for 3D non-crimp fabrics (NCF) and double-bar knitting for spacer fabrics.

For 3D NCF, there are approaches at material, structure and process level to improve the pre-forming of NCF in terms of wrinkle-free drape without overlap. It should be noted here that the structural modification of NCF has the potential to enable not only wrinkle- and overlap-free draping, but also load-path-compatible filament orientation through the targeted introduction of yarn reserves in the manufacturing process. However, this has not yet been implemented, as the focus so far has been on geometry-appropriate draping and the corresponding generation of yarn reserves.

In the case of warp knitted spacer fabrics, it is also possible to optimise the appearance of the spacer fabric for a specific application by varying the material, structure and process. This can reduce the number of finishing operations and therefore costs and waste. In addition, the research results researched show that application-specific performance can be increased (e.g., in the form of tubular spacer fabrics) and that completely new textile materials open up completely new fields of application (e.g., thermogenerators or spacer fabrics in combination with 3D printing elements).

In order to contribute to an increase in the industrial application of 3D warp knitted fabrics through targeted further development, it is necessary to have an overview of the existing technologies, to which this review contributes.

## Data Availability

Data is contained within the article.
